# Alpha-Ketoglutarate Regulates Tnfrsf12a/Fn14 Expression via Histone Modification and Prevents Cancer-Induced Cachexia

**DOI:** 10.3390/genes14091818

**Published:** 2023-09-19

**Authors:** Bryan I. Ruiz, Xazmin H. Lowman, Ying Yang, Qi Fan, Tianhong Wang, Hongmei Wu, Eric A. Hanse, Mei Kong

**Affiliations:** Department of Molecular Biology and Biochemistry, School of Biological Sciences, University of California, Irvine, CA 92697, USA

**Keywords:** cancer, cachexia, colon cancer, FN14, *TNFRSF12A*, alpha-ketoglutarate, histone

## Abstract

Previous studies have shown that inhibition of TNF family member FN14 (gene: *TNFRSF12A*) in colon tumors decreases inflammatory cytokine expression and mitigates cancer-induced cachexia. However, the molecular mechanisms underlying the regulation of FN14 expression remain unclear. Tumor microenvironments are often devoid of nutrients and oxygen, yet how the cachexic response relates to the tumor microenvironment and, importantly, nutrient stress is unknown. Here, we looked at the connections between metabolic stress and FN14 expression. We found that *TNFRSF12A* expression was transcriptionally induced during glutamine deprivation in cancer cell lines. We also show that the downstream glutaminolysis metabolite, alpha-ketoglutarate (aKG), is sufficient to rescue glutamine-deprivation-promoted *TNFRSF12A* induction. As aKG is a co-factor for histone de-methylase, we looked at histone methylation and found that histone H3K4me3 at the *Tnfrsf12a* promoter is increased under glutamine-deprived conditions and rescued via DM-aKG supplementation. Finally, expression of *Tnfrsf12a* and cachexia-induced weight loss can be inhibited in vivo by DM-aKG in a mouse cancer cachexia model. These findings highlight a connection between metabolic stress and cancer cachexia development.

## 1. Introduction

FN14 is a type 1 transmembrane protein with the gene name tumor necrosis factor receptor superfamily member 12A (*TNFRSF12A*). Previous studies have found the transcription of FN14 in a variety of tissues, such as the liver, heart, lung, and skeletal muscle [1,2,3,4]. FN14 is also found to be upregulated across a variety of high-grade solid tumors, including glioma, pancreatic, breast, non-small-cell lung cancer, and colorectal cancer (CRC) [5,6,7,8,9]. Importantly, it has been previously shown that specific inhibition of FN14 in CRC is able to decrease inflammatory cytokine expression as well as cancer-induced cachexia [10].

Cancer cachexia is defined as a multifactorial syndrome characterized by the progressive loss of skeletal muscle mass that cannot be entirely reversed with nutritional support [11]. As cachexia progresses, patient organs display a net negative energy and protein balance driven by a variable combination of reduced food intake and abnormal metabolism induced by the tumor. On the other hand, tumor cells often reprogram their metabolism to meet the increased requirement of rapid proliferation. For example, in colorectal cancer (CRC), it has been indicated that glutamine (Gln) is scavenged from muscle tissue to support cancer cell metabolism with increased expression of glutaminase [12,13]. Cancer cells use Gln in order to sustain proliferation and cell growth specifically by using Gln as a carbon source to drive the TCA during Warburg metabolism [14]. Moreover, Gln is used by proliferating cells to generate nucleotides, and glutathione is used to modulate ROS [15,16]. However, as a result of unchecked biosynthesis and outgrowth of vascular supply lines, the tumor is eventually unable to sustain intratumoral concentrations of Gln, leading tumor cells into a glutamine-deprived environment [17,18,19]. This phenomenon occurs across multiple cachexic cancer types as several studies show that solid tumors, such as colorectal cancer, melanoma, and pancreatic cancer, reside in glutamine-deprived tumor microenvironments (TME) [18,20,21]. However, how this tumor metabolic environment affects cancer-induced cachexia is largely unknown.

Alpha-ketoglutarate (aKG), an intermediate metabolite found two steps downstream from Gln during the process of glutaminolysis, has been shown to control gene expression by regulating the epigenetic architecture in normal and cancer cells [22,23]. Specifically, depletion of aKG from the TME has been shown to increase global histone methylation as a result of reduced activity of Jumonji histone demethylases (JHDMs), which use aKG as a cofactor [23]. In turn, this fosters hypermethylation of H3K4me3, a marker for active transcription [23,24,25]. This hypermethylated phenotype promotes cancer dedifferentiation via Wnt signaling, increases cancer cell survival, and upregulates systemic inflammatory pathways including VEGF, IL-1, and IL-8 within tumors [23]. Notably, this is reversible through supplementation of Gln or aKG in mice, and results in an increase in mice survival and a decrease in tumor burden [23,24]. Additionally, the supplementation of aKG and Gln have both been used in the treatment of cachexia-associated phenotypes. For example, aKG has been seen to reduce skeletal muscle protein degradation within the context of Duchenne muscular dystrophy, and Gln supplementation has been shown to both reduce loss of body mass as well as increase muscle protein synthesis in cancer cachexia [26,27,28]. Despite the efficacy of aKG and Gln supplementation in the prevention of cachexia within mice and rats, the mechanisms underlying the prevention of cancer-associated cachexia are less understood.

In this study, we show that FN14 expression is significantly upregulated in response to Gln deprivation across a variety of cancer cell lines. This effect is rescued by dimethyl alpha-ketoglutarate (DM-aKG) supplementation. These results provide support for therapeutic strategies that sustain Gln concentrations within the tumor microenvironment as a means of regulating FN14 expression and, subsequently, cachexia.

## 2. Materials and Methods

### 2.1. Cell Culture and Media

SW620, CT26, and PANC-1 cell lines were purchased from ATCC. Mouse-derived C26 cells were graciously donated by Dr. Andrea Bonnetto of Indiana State University. Gln-deprived and Gln-supplemented media were made with DMEM devoid of Gln and sodium-pyruvate (Corning, New York, NY, USA) supplemented with L-Glutamine (Omega Scientific, Tarzana, CA, USA), 10% dialyzed fetal bovine serum (Omega Scientific, Tarzana, CA, USA), and penicillin/streptomycin (Genesee Scientific, Morrisville, NC, USA). Cells were cultured at 37 °C with 5% CO_2_. Within Dulbecco’s MEM (DMEM) media, Dimethyl alpha-ketoglutarate (Tokyo Chemical Industry, Tokyo, Japan), Succinate (Sigma Aldrich, St. Lous, MO, USA), N-acetylcysteine (Sigma Aldrich, St. Lous, MO, USA), dialyzed fetal bovine serum (Omega Scientific, Tarzana, CA, USA), and D-Glucose (Sigma Aldrich, St. Lous, MO, USA) were supplemented as indicated. For hypoxia culture, cells were seeded in DMEM with 10% dialyzed FBS and 0.2 mM or 4 mM glutamine under 1% O_2_ using a BioSpherix Xvivo system (BioSpherix, Parish, NY, USA). The cells were maintained under hypoxic conditions for 24 h until collection. For amino acid deprivation experiments, DMEM with Low Glucose, *w*/*o* Amino Acids, Pyruvic Acid (Powder) (US Biological, Salem, MA, USA) was used. Nonessential amino acids (Sigma Aldrich, St. Lous, MO, USA), branched-chain amino acids (Sigma Aldrich, St. Lous, MO, USA), and essential Amino acids (Sigma Aldrich, St. Lous, MO, USA) were added according to the DMEM formulations found in the Table 1. 

### 2.2. RNA Extraction and Quantitative Real-Time PCR

Total RNA was extracted and purified using Trizol according to the manufacturer’s instructions (Life Technologies, Carlsbad, CA, USA). Complementary DNA (cDNA) was made using 1 μg of RNA and the qScript cDNA Synthesis Kit (Quanta BioSciences, Beverly, MA, USA). Quantitative real-time PCR (qPCR) was performed using the SYBR Green PCR Master Mix (Quanta BioSciences, Beverly, MA, USA) on a Bio-Rad real-time PCR machine. Relative gene expression was calculated using the 2^–∆∆Ct^ method and normalized to 18S or Actin RNA. The primers used in the study are shown in Table 2.

### 2.3. Flow Cytometry

Cells were washed once with cold PBS and trypsinized (Gibco, Grand Island, NY, USA). Cells measuring 2 × 10^6^ were resuspended in fluorescence-activated cell sorting (FACS) staining buffer (FSB) (2% fetal bovine serum and 0.01% NaN3) for 20 min at 4 °C with 0.5 μg antibodies or isotype-matched IgG. Cells were then washed twice with FSB and fixed with FACS fixative (1% formaldehyde in PBS). Antibodies used were FN14 (CD266, eBioscience, Santa Clara, CA, USA) and Mouse IgG2b kappa Isotype Control (eBMG2b), PE, (eBioscience, Santa Clara, CA, USA). A total of 100,000 events were collected per sample. Flow analysis was conducted using a BD Fortessa X20 (BD Biosciences, San Jose, CA, USA). 

### 2.4. Histone Extraction and Western Blot

Cells were washed twice with PBS and lysed with hypotonic lysis buffer with protease inhibitors (10 mM HEPES, 10 mM KCl, 1.5 mM MgCl_2_, 0.5 mM dithiothreitol, and Protease inhibitor (Roche, Mannheim, GER) on ice for 1 h. The lysate was then rotated overnight at 4 °C in a final concentration of 0.2 N H_2_SO_4_. The samples were then centrifuged, supernatants were collected, and histones were precipitated for 1 h on ice using 25% (*w*/*v*) TCA. The pellet was then washed three times with ice-cold acetone and dissolved in water. Membranes were blocked for 1 h in 5% non-fat milk in PBS with 0.05% Tween-20 (Sigma Aldrich) and incubated with antibodies overnight. Membranes (Bio-Rad) were visualized using horseradish-peroxidase-conjugated secondary antibodies (1:2000, Bio-Rad, Hercules, CA, USA) and Western Lightning Plus-ECL (PerkinElmer, Waltham, MA, USA). The immunoblots were normalized against Histone H3 using ImageJ. Antibodies used for the study include H3K4me3 (EMD Millipore, Burlington, MA, USA) at 1:1000 and H3 (Cell Signaling, Danvers, MA, USA) at 1:12,000.

### 2.5. Chromatin Immunoprecipitation Assay

The chromatin immunoprecipitation assay (ChIP) was performed using the ChIP assay kit (EMD Millipore, Burlington, MA, USA) according to the manufacturer’s guidelines. The cells were cultured in Gln-supplemented (4 mM) or Gln-deprived media with or without DM-aKG (4 mM) for 24 h. The cells were then crosslinked with 1% formaldehyde for 10 min at 37 °C. Cells were then washed twice with PBS containing protease inhibitors (Roche, Mannheim, GER) and lysed with SDS lysis buffer. The cell lysates were then sonicated to yield 200–1000 bp DNA fragments. The cell lysates were then centrifuged and supernatants were diluted with immunoprecipitation buffer with protease inhibitors (Roche, Mannheim, GER). The dilutant was then immunoprecipitated with 1 μg of H3K4me3 antibody (EMD Millipore, Burlington, MA, USA) or IgG overnight at 4 °C with rotation. Chromatin DNA was obtained according to the manufacturer’s specifications. The DNA was then analyzed via qPCR with the primers shown in Table 3. 

### 2.6. Mouse Intestinal Crypt Isolation and Organoid Culture 

Intestinal crypts from 6–8-week-old *Apc*^Min/+^ mice and wild-type mice (Jackson Laboratory, Bar Harbor, ME, USA) were isolated from tumor-free small intestinal tissues based on a previous study [29]. Crypts were cultured under IntestiCult™ organoid media supplemented with EGF, Noggin, and R-Spondin (Stemcell), and Primocin mixed 1:1 with Growth-Factor-Reduced Matrigel (Corning). An amount of 50 μL of the mixture was plated in a prewarmed 48-well plate and incubated for 10 min at 37 °C. For Gln deprivation experiments, Gln-free DMEM/F-12 (Gibco) was supplemented with L-Glutamine (Corning) or Dimethyl alpha-ketoglutarate (Tokyo Chemical Industry, Tokyo, Japan) as indicated.

### 2.7. Animal Models and Xenograft

All studies utilizing animals were performed according to the protocol approved by the Institutional Animal Care and Use Committee (IACUC) at the University of California, Irvine, in compliance with ethical regulations. CD2F1 (Charles River) male mice aged 10-weeks-old were used for xenograft studies. For the xenograft, mice were injected subcutaneously with 0.5 × 10^6^ C26 cells in 200 μL DMEM (Corning) devoid of FBS or antibiotics. Mice were placed randomly into two groups one-week post engraftment. Mouse weight, tumor length, and width were measured every 2–4 days using calipers; tumor volume was then calculated using the formula ½ (length × width^2^). Mice were euthanized upon 15% body mass loss, signs of ulceration/bleeding, or signs of lethargy. DM-aKG at 600 mg/kg or PBS was delivered intraperitoneally every 2–4 days beginning when tumor volume was at a minimum of 100 mm^3^. Estimated sample sizes for each animal study were based on preliminary data or prior experience with these models, anticipating group variance.

### 2.8. Statistics 

Unpaired *t*-test and ordinary one-way ANOVA with post hoc Tukey’s test or Sidak’s correction were used to assess the statistical significance of mean differences and calculated using GraphPad Prism (v9) software. To determine the *p* values of DM-aKG versus PBS interpretational injection, ordinary one-way ANOVA with post hoc Tukey’s test was applied. For all statistics: ns represents *p* > 0.05, * represents *p* ≤ 0.05, ** represents *p* ≤ 0.01, *** represents *p* ≤ 0.001, and **** represents *p* ≤ 0.0001.

## 3. Results

### 3.1. Glutamine Deprivation Induces TNFRSF12A Expression

**Figure 1:**
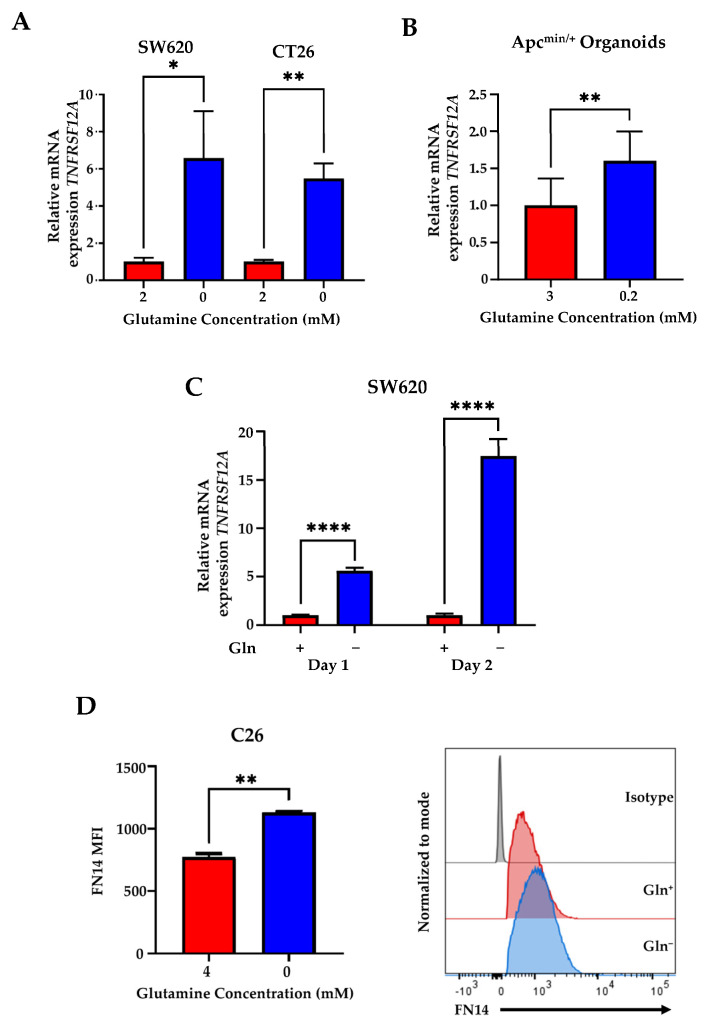
Glutamine deprivation induces *TNFRSF12A* expression. (**A**) qPCR analysis of *TNFRSF12A* in human colorectal cancer cells SW620 and mouse CT26 cells cultured in glutamine (Gln)-deprived medium (0 mM) or 2 mM Gln for 24 h. (**B**) qPCR analysis of *Tnfrsf12a* in mouse ApcMin/+ organoids cultured in low-Gln medium (0.2 mM) or 3 mM Gln for 72 h. (**C**) qPCR analysis of *Tnfrsf12a* in SW620 cultured in low-Gln medium (0.2 mM) or 4 mM Gln for 24 h. (**D**) Mean fluorescence intensity (MFI) of anti-FN14 and representative histogram of FN14 protein expression levels in C26 cells cultured in Gln-deprived medium (0 mM) or 4 mM Gln for 24 h (*n* = 2). Data represent the mean ± SD (*n* = 3) unless specified. * *p* ≤ 0.05, ** *p* ≤ 0.01, **** *p* ≤ 0.0001.

*TNFRSF12A* or FN14 is thought to be sufficient for cachexia and a link has been made between *TNFRSF12A* and CRC [8,10]. Additionally, it has been shown that tumor cells can adapt to Gln deprivation by altering gene expression [18,23,24]. To examine the effects of glutamine deprivation on *TNFRSF12A* CRC cell lines, human SW620 and mouse CT26 were Gln-deprived and the expression of *TNFRSF12A* was assayed via qPCR. We found that glutamine deprivation induces *TNFRSF12A* expression in both cell lines (Figure 1A). To confirm this induction, we further examined *Tnfrsf12a* expression in *Apc*^Min/+^ organoids under low Gln conditions, a system that highly resembles the in vivo profile of CRC. We found that glutamine deprivation significantly induces *Tnfrsf12a* expression in organoids (Figure 1B). Previous studies in glioma have shown that FN14 displays transient upregulation under serum-deprived conditions and TWEAK co-stimulation but decreases within 8 h [9]. We thus wanted to determine if Gln deprivation was able to sustain *TNFRSF12A* expression during Gln deprivation as CRC are chronically in a state of low Gln concentrations. As such, SW260 cells were cultured under low Gln conditions for 24 and 48 h. We found that *TNFRSF12A* expressions increased 5.6-fold over 24 h and further increased to 17.49-fold over 48 h (Figure 1C). These results show that Gln deprivation induces *TNFRSF12A* transcription, and this effect is sustained over time. Next, we asked whether induced *TNFRSF12A* expression results in increased FN14 protein expression. Using flow-cytometry-based analysis, we confirmed that the FN14 protein expression is also induced upon glutamine deprivation (Figure 1D). 

### 3.2. TNFRSF12A Transcriptional Induction Is Specific to Glutamine Deprivation

**Figure 2:**
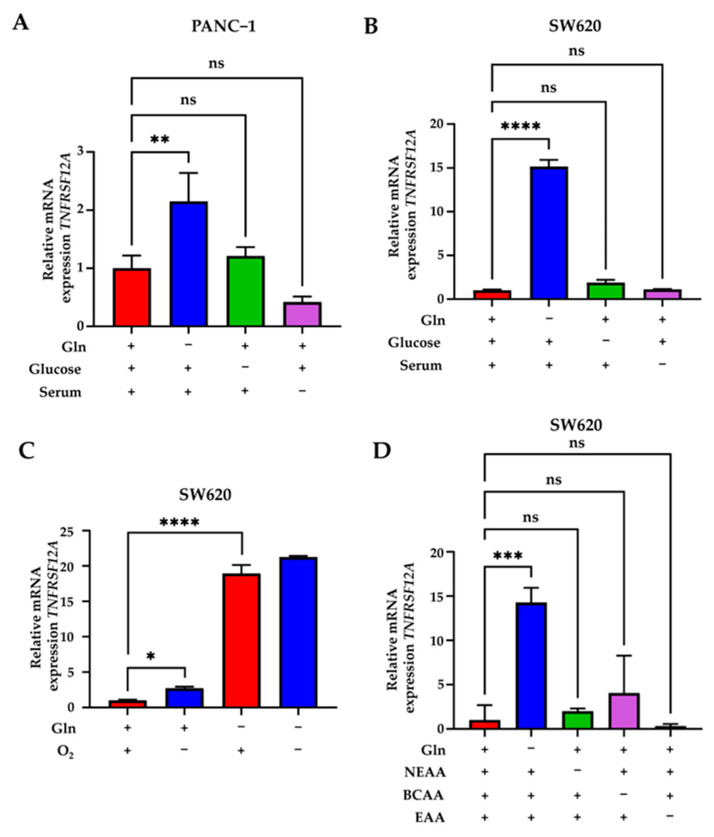
*TNFRSF12A* transcriptional induction is specific to glutamine deprivation. (**A**) qPCR analysis of *TNFRSF12A* in PANC1 cells cultured in 0 mM glutamine medium (Gln) (−) or 4 mM Gln (+) with either 4.5 g/L glucose (+), no glucose (−), 10% serum (+), or 1% serum (−). (**B**) qPCR analysis of *TNFRSF12A* in SW620 cells cultured in 0 mM Gln (−) or 4 mM Gln (+) with either 1.0 g/L glucose (+), no glucose (−), 10% serum (+), or 1% serum (−). (**C**) qPCR analysis of *TNFRSF12A* in SW620 cultured in 0.2 mM Gln medium (−) or 4 mM Gln (+) under 1% O2 (−) or 20% O2 (+) for 24 h. (**D**) qPCR analysis of *TNFRSF12A* in SW620 cultured in 0 mM Gln medium (−) or 4 mM Gln or media with (+) or without (−) nonessential amino acids (NEAAs), branched-chain amino acids (BCAAs), or essential amino acids (EAAs). Data represent the mean ± SD (*n* = 3). ns *p* > 0.05, * *p* ≤ 0.05, ** *p* ≤ 0.01, *** *p* ≤ 0.001, **** *p* ≤ 0.0001.

As FN14 is over-expressed in a variety of tumors, such as CRC and pancreatic cancer, and nutrient deprivation is a common characteristic of the tumor microenvironment [8,21,30], we further asked if FN14 induction is specific to glutamine deprivation using different cancer lines under a few different metabolic stress conditions. Pancreatic cancer PANC-1, a cachexia-inducing cell line [31], and CRC SW620 cells were deprived of either Gln, glucose, or serum for 24 h, and the expression of *TNFRSF12A* mRNA was measured via qPCR. We found that only glutamine deprivation significantly induced *TNFRSF12A* expression in both cell lines (Figure 2A,B). FN14 expression was previously found to be upregulated in contexts of hypoxia [32]. Similarly, we found that hypoxia significantly induced *TNFRSF12A* mRNA expression. However, Gln deprivation induced *TNFRSF12A* expression and was found to be significantly higher than the hypoxia condition (Figure 2C). In addition, hypoxia cannot further promote Gln-deprivation-induced *TNFRSF12A* expression (Figure 2C). We also found *TNFRSF12A* mRNA expression is specific to Gln starvation as removal of non-essential amino acids, essential amino acids, and branch chain amino acids from media are unable to induce *TNFRSF12A* transcription (Figure 2D). Taken together, these results show Gln deprivation has a more pronounced effect in inducing *TNFRSF12A* expression compared with other metabolic stresses. 

### 3.3. Dimethyl Alpha-Ketoglutarate Supplementation Is Sufficient to Inhibit TNFRSF12A Expression In Vitro

**Figure 3:**
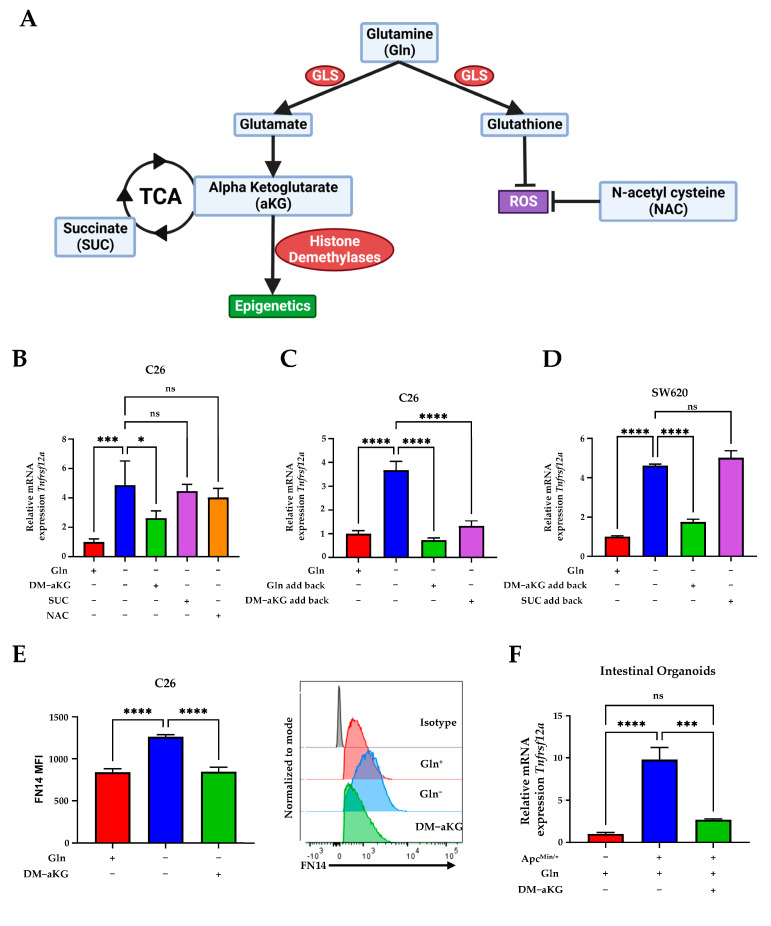
Dimethyl alpha-ketoglutarate supplementation is sufficient to inhibit *TNFRSF12A* expression in vitro. (**A**) Schematic diagram of glutamine metabolism created with BioRender. (**B**) qPCR analysis of *Tnfrsf12a* in C26 cells cultured in conditions as indicated: 0 mM Gln (−), 4 mM Gln (+), 4 mM Dimethyl alpha-ketoglutarate (DM-aKG) (+), 5 mM N-acetylcysteine (NAC) (+), or 5 mM Succinate (SUC) (+). (**C**) qPCR analysis of *Tnfrsf12a* in C26 cells cultured in 0 mM Gln for 16 h. An amount of 2 mM Gln (+) or 2 mM DM-aKG (+) were then added back into Gln-deprived media for 8 h. (**D**) qPCR analysis of *TNFRSF12A* in SW620 cells cultured in 0 mM Gln for 16 h. An amount of 2 mM Gln (+), 4 mM Dm-aKG (+), or 5 mM SUC (+) were then added back into Gln-deprived media for 8 h. (**E**) Mean fluorescence intensity (MFI) of anti-FN14 and representative histogram of FN14 protein expression levels in C26 cells cultured in 0 mM Gln, 4 mM Gln, or 4 mM DM-aKG for 24 h. (**F**) qPCR analysis of *Tnfrsf12a* in wild type (−) or *Apc*^Min/+^ (+) organoids cultured in low-Gln medium (0.2 mM) (−) or 3 mM Gln (+) for 72 h. For all experiments, data represent the mean ± SD (*n* = 3). ns *p* > 0.05, * *p* ≤ 0.05, *** *p* ≤ 0.001, **** *p* ≤ 0.0001.

Once transported into the cell, Gln is broken down quickly by glutaminolysis into Glutamate (Glu) and then aKG which is not only a critical TCA component but can also serve as a co-factor for histone modification enzymes (Figure 3A). Specifically, aKG serves as a cofactor for the function of Jumonji histone demethylases and TET (ten–eleven translocation) methylcytosine dioxygenases [33,34]. Additionally, Gln can produce glutathione (GSH), which can be used to control redox conditions [35,36] (Figure 3A).

To ask if depleted Gln/Glutaminolysis and TCA byproducts are key effectors of *Tnfrsf12a* expression, CRC cells were deprived of Gln but supplemented with either DM-aKG, succinate (SUC), or antioxidant N-acetyl-L-cysteine (NAC). Interestingly, we found that a cell-permeable DM-aKG was able to rescue Gln-deprivation-induced *Tnfrsf12a* transcription, whereas supplementation with either SUC or NAC was unable to rescue *Tnfrsf12a* mRNA upregulation (Figure 3B). Next, to test if DM-aKG can reverse Gln-deprivation-induced *Tnfrsf12a* transcription, we cultured C26 or SW620 cells in Gln-free media for 16 h to induce *Tnfrsf12a* expression followed by the addback of glutamine, DM-aKG, or SUC. We found that addback with DM-aKG significantly suppressed *Tnfrsf12a* expression (Figure 3C,D). In addition, we found that DM-aKG was able to suppress FN14 protein expression in Gln-deprived culture (Figure 3E). Additionally, to test if *Tnfrsf12a* upregulation is relevant to CRC, we examined *Tnfrsf12a* expression in WT or *Apc*^Min/+^ mutant intestinal organoids and found that *Tnfrsf12a* expression is significantly higher in *Apc*^Min/+^ organoids. Additionally, supplementation of DM-aKG was able to decrease the expression of *Tnfrsf12a* in *Apc*^Min/+^ organoids (Figure 3F). Taken together, our findings show the expression of *Tnfrsf12a* can be suppressed by DM-aKG.

### 3.4. Dimethyl Alpha-Ketoglutarate Regulates Tnfrsf12a Expression via Histone Modification of H3K4me3

**Figure 4:**
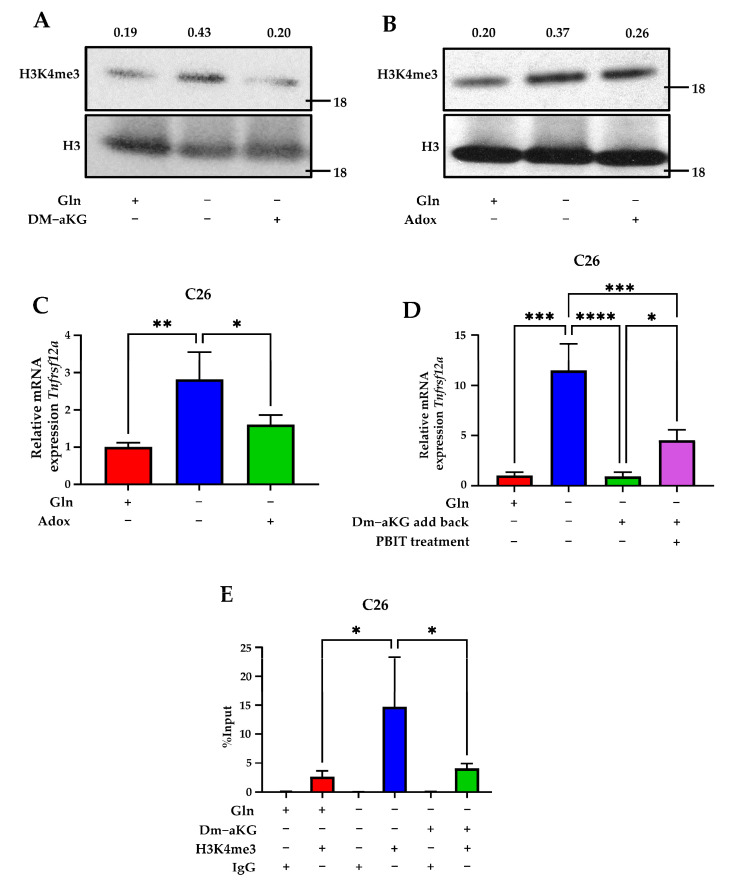
*Tnfrsf12a* expression is regulated by histone modification of H3K4me3. (**A**) Western blot of H3K4me3 in C26 cells cultured in 0.1 mM glutamine (Gln) (−), 2 mM Gln (+), or (+) 4 mM dimethyl alpha-ketoglutarate (DM-aKG) (+) annotated with band density relative to H3. (**B**) Western blot of H3K4me3 in C26 cells cultured in 0.1 mM Gln (−) and 2 mM Gln (+), with (+) or without (−) 50 µM Adox annotated with a band density relative to H3. (**C**) qPCR analysis of *Tnfrsf12a* in C26 cells cultured in 0 mM Gln (−) and 4 mM Gln (+), with (+) or without (−) 50 µM Adox. (**D**) qPCR analysis of *Tnfrsf12a* in C26 cells cultured in 0 mM Gln medium for 16 h. An amount of 4 mM Gln (+), 4 mM Dm-aKG (+), or 5 µM PBIT (+) were then added back into Gln-deprived media for 8 h. (**E**) ChIP analysis of H3K4me3 levels on the promoter region of *Tnfrsf12a* in C26 cells cultured in 0 mM Gln (−), 4 mM Gln (+), and 4 mM DM-aKG (+). Data represent the mean ± SD (*n* = 3). * *p* ≤ 0.05, ** *p* ≤ 0.01, *** *p* ≤ 0.001, **** *p* ≤ 0.0001.

Previous data show that during Gln deprivation CRC undergoes global histone hypermethylation of H3K4me3 due in part to the loss of aKG and ability to activate histone demethylases [23,24]. This, in turn, results in increased oncogene expression. Upon treatment with either Gln or aKG, H3K4me3 methylation can be suppressed and leads to decreased expression of many oncogenes [23,24]. To test if *Tnfrsf12a* is regulated by histone demethylase activity, the methylation of H3K4me3 was probed in C26 cells upon Gln starvation and/or DM-aKG supplementation. As previously shown, H3K4me3 was found to increase during Gln starvation which is rescued by DM-aKG supplementation (Figure 4A). To test if *Tnfrsf12a* is regulated by histone demethylase activity, C26 cells were treated with adenosine dialdehyde (Adox), a global methyltransferase inhibitor [37,38]. We found that under Gln-deprived conditions, Adox was able to decrease the expression of *Tnfrsf12a*, indicating that transcriptional induction of *Tnfrsf12a* is in part through the regulation of methyltransferase activity (Figure 4B,C). Since the JARID1 family of JHDMs regulates the methylation of H3K4me3, we next tested to see if the JARID1 family serves to regulate the expression of *Tnfrsf12a*. PBIT is a more selective inhibitor than Adox that targets only members of the demethylases belonging to the JARID1 (Jumonji) family [39]. To verify if the expression of *Tnfrsf12a* is regulated by JARID1 family demethylases at H3K4, C26 cells were deprived of Gln for 16 h; afterward, DM-aKG or DM-aKG with 5 µM PBIT were added, and cells were collected after 8 h. We found that addback of DM-aKG with 5 µM PBIT resulted in a partial but significant rescue of *Tnfrsf12a* transcription (Figure 4D). To assess whether the *Tnfrsf12a* promoter region was regulated by H3K4me3, we performed chromatin immunoprecipitation (ChIP) of H3K4me3 and assessed for the *Tnfrsf12a* promoter region via qPCR. ChIP-qPCR of the *Tnfrsf12a* promoter region revealed that glutamine deprivation increases the extent of H3K4me3 at the *Tnfrsf12a* promoter and this effect can be mitigated via DM-aKG supplementation (Figure 4E). Together, our results show that H3K4me3 methylation increases at the *Tnfrsf12a* promoter under glutamine-deprived conditions and this effect is partly facilitated by JARID1 methyltransferase activity.

### 3.5. Dimethyl Alpha-Ketoglutarate Supplementation Is Sufficient to Rescue Cachexia and Tnfrsf12a Transcription In Vivo

**Figure 5:**
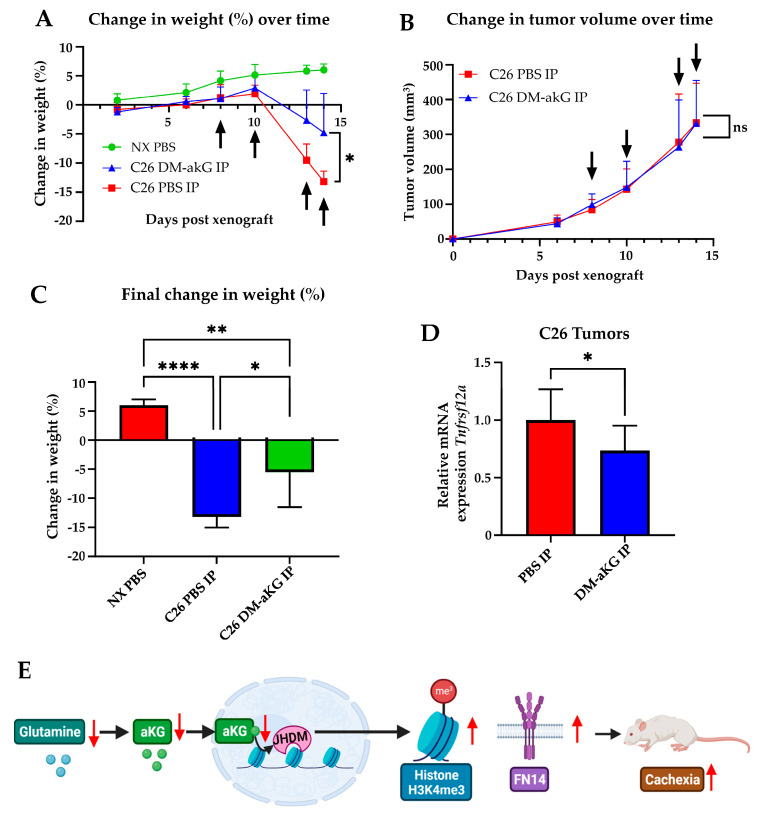
Dimethyl alpha-ketoglutarate supplementation is sufficient to rescue *Tnfrsf12a* transcription and cachexia in vivo. (**A**) CD2F1 mice with subcutaneous injection of C26 cells. Dimethyl alpha-ketoglutarate (DM-aKG) 600 mg/kg or PBS was delivered intraperitoneally every 2–4 days (arrows). Weight was measured every 2–4 days (non-xenograft (NX) PBS, *n* = 4; C26 PBS IP and C26 DM-aKG IP, *n* = 5). (**B**) Tumor volume was measured every 2–4 days (NX PBS, *n* = 4; C26 PBS IP and C26 DM-aKG IP *n* = 5). (**C**) Final weight change from initial weight of CD2F1 mice with NX or C26 cells intraperitoneally injected with PBS or DM-aKG. (**D**) qPCR analysis of the DM-aKG- (C26 DM-aKG IP, *n* = 3) or PBS- (C26 PBS IP, *n* = 4) treated tumors. (**E**) Schematic of the proposed mechanism created with BioRender. ns *p* > 0.05, * *p* ≤ 0.05, ** *p* ≤ 0.01, **** *p* ≤ 0.0001.

Previous data show that inhibition of tumor-derived Fn14 is sufficient to perturb cachexia [10]. We then wanted to examine whether DM-aKG delivery via intraperitoneal injection was capable of blocking tumor-induced cachexia in vivo. C26 cells were xenografted into CD2F1 mice, an established CRC cachexia mouse model. We found that intraperitoneal (IP) injection of DM-aKG was able to decrease cachexia compared with PBS, respectively amounting to −5.48% versus −13.19% body mass loss over the course of 14 days without influencing tumor volume during that same duration (Figure 5A–C). However, if treatment was continued past 14 days, DM-aKG IP is capable of decreasing tumor volume growth as previously described [23]. Concurrently, DM-aKG treatment was sufficient to inhibit *Tnfrsf12a* transcription in the primary tumor (Figure 5D). Our findings establish a novel relationship between Gln deprivation that leads to histone methylation increases, and the induction of the cachexia-promoting factor FN14. As modeled in Figure 5E, the Gln-deficient condition promotes global H3K4me3 histone methylation due to decreased JHDM activity, which sustains the H3K4me3 methylation and upregulates *Tnfrsf12a* and FN14. Further, FN14 expression has been shown to promote a cachectic, wasting phenotype. We found this process can be rescued in vivo by supplementing DM-aKG, which mitigates cachexia-induced weight loss.

## 4. Discussion

Mechanisms describing the regulation of FN14 include transcription factor ThPOK, cytokines, DNA methylation, and miRNAs, as well as physical cellular stress [5,40,41,42,43,44]. Our investigations across a variety of cancerous cell lines and *Apc*^Min/+^ organoids have shown that metabolic stress in the form of Gln deprivation is a major contributor to the regulation of *Tnfrsf12a*. We show that the upregulation of *Tnfrsf12a* is independent of the other major metabolic stressors known to affect cancer metabolism such as glucose and serum deprivation (Figure 2A,B). We also found that other amino acids could not rescue what glutamine deprivation achieves on its own (Figure 1A,B and Figure 2D). Interestingly, ROS activation of FN14 has been shown and *Tnfrsf12a* contains conserved hypoxia-inducible factor (HIF)-1α binding sites in retinal neovascularization models [32]. However, we found that supplementing with one dose of NAC was unable to consistently prevent *Tnfrsf12a* induction under glutamine deprivation (Figure 3A). Future experiments need to be performed with different doses and time periods to achieve a conclusion. Instead, our results suggest that Gln deprivation at the site of the tumor decreases the concentration of aKG, which stimulates *Tnfrsf12a* transcription as a form of metabolic adaptation that calls to distant sites for nutrients in the form of cachexia. Although *TNFRSF12A* expression increased in human tumors [5,6,7,8,9], especially those with high cachexia incidence, a correlation between *TNFRSF12A* levels with weight loss in patients has not been established due to lacking information concerning the presence of cachexia in current patient databases. It is interesting to note that the TCGA database analysis promisingly revealed positive correlations between many inflammatory cytokines implicated in cancer cachexia and Fn14 transcripts in patients [10].

We have previously shown that histone hypermethylation is induced in CRC tumors as a consequence of Gln deprivation [23]. Specifically, deficiency of Gln from the tumor microenvironment was shown to result in the methylation of H3K4, a marker for active transcription [24]. We also found that the mechanism of transcriptional induction of *Tnfrsf12a* is due in part to the decreased activity of JDHMs brought on by Gln deprivation. Further, the induction of *Tnfrsf12a* could only be rescued by the introduction of cell-permeable DM-aKG or Gln; the former serves as a cofactor for JDHMs, whereas the latter can be metabolized into aKG. Using the JARID1-specific inhibitor PBIT, we specifically pinpointed the JARID1 histone demethylases as regulators capable of decreasing the expression of *Tnfrsf12a*. These results were further corroborated by ChIP-qPCR of the H3K4me3, where in Gln-deprived conditions it showed increased H4K4 methylation at the *Tnfrsf12a* promoter, which was then decreased by DM-aKG supplementation. Interestingly, our study is not the first to discern a mechanism for the activation of FN14 regulated by epigenetic mechanisms in chronic disease states capable of inducing cachexia. Tajrishi et al. found that muscular denervation diminishes overall genomic DNA methylation and causes hypomethylation of *TNFRSF12A* at its CpG-rich promoter region via methyltransferase DMNT3A, thus facilitating its activation [4]. This finding is particularly interesting as previous data have shown that DNA CpG islands correlate closely with H3K4me3 methylation [45,46,47]. Additionally, the ability of FN14 to be induced under various contexts of global methylation may also account for its overexpression within the context of gliomas which are characteristically Gln-rich and Gln-addicted [9,48,49].

Furthermore, we found that the rescue of *Tnfrsf12a* transcription via DM-aKG was possible both in vitro as well as in vivo. In that, IP injection of DM-aKG was able to decrease the extent of body mass loss within mice, indicating that DM-aKG treatment may serve as an important avenue in the treatment of cachexia. Previous preclinical studies have already touched on the importance of aKG treatment in preventing skeletal muscle protein degradation [26,50,51]. However, sustaining subject weight using aKG in preclinical studies has proven difficult as studies using Ornithine alpha-ketoglutarate in the context of cancer-induced cachexia were unable to prevent body mass loss [50]. Our positive results may be the result of using cell-permeable DM-aKG, which may make delivery into the tumor and peripheral tissues more efficient. In summary, our findings establish a novel connection between Gln deprivation and the induction of the cachexia-inducing factor FN14. Our results show that under Gln-deprived conditions, global histone methylation of H3K4me3 increases, resulting in an upregulation of *Tnfrsf12a* and cachexia. This effect can be rescued both in vivo and in vitro through the introduction of DM-aKG, resulting in mitigation of cachexia-induced weight loss and FN14 expression. Ultimately, our results provide evidence for further exploration of aKG as a therapeutic strategy for cancer cachexia.

## Figures and Tables

**Table 1:** Amino Acid deprivation working concentrations.

Nonessential Amino Acids (NEAAs)		Essential Amino Acids (EAAs)		Branched-Chain Amino Acids (BCAAs)	
Metabolite	Conc. (mM)	Metabolite	Conc. (mM)	Metabolite	Conc. (mM)
Glycine	0.4	L-Lysine·HCl	0.8	L-Isoleucine	0.8
L-Arginine·HCl	0.4	L-Methionine	0.2	L-Leucine	0.8
L-Cystine·2HCl	0.2	L-Phenylalanine	0.4	L-Valine	0.8
L-Serine	0.4	L-Histadine·HCl	0.2		
L-Tyrosine disodium salt dihydrate	0.4	L-Threonine	0.8		
		L-Tryptophan	0.08		
**Media Metabolite Supplementations:**					
NEAA-deprived media			EAA only		
EAA-deprived media			NEAA only		
BCAA-deprived media			EAA + NEAA only		

**Table 2:** Primers used in the study for qPCR.

Title	Sequence	Figure
m-*Tnfrsf12a* (FWD)	5′-GTGTTGGGATTCGGCTTGGT-3′	Figures 1A,B, 3B,C, 4C,D and 5D
m-*Tnfrsf12a* (REV)	5′-GGCAGAAGTCGCTGTGTGGT-3′	Figures 1A,B, 3B,C, 4C,D and 5D
m-*Tnfrsf12a* (FWD)	5′-GCAGATCCTCGTGTTGGGAT-3′	Figure 3F
m-*Tnfrsf12a* (REV)	5′-GGACAAGAAGCGCAGTCCAT-3′	Figure 3F
h-*TNFRSF12A* (FWD)	5′-CTGGCTCCAGAACAGAAAGG-3′	Figure 1A
h-*TNFRSF12A* (REV)	5′-GGGCCTAGTGTCAAGTCTGC-3′	Figure 1A
h-*TNFRSF12A* (FWD)	5′-TTT GGT CTG GAG ACG ATG CC-3′	Figures 1C, 2A–D and 3D
h-*TNFRSF12A* (REV)	5′-TGAATGATGAGTGGG CGA GC-3′	Figures 1C, 2A–D and 3D
m-*18s* (FWD)	5′-CGCTTCCTTACCTGGTTGAT-3′	Figures 1A,B, 3B,C,F, 4C,D and 5D
m-*18s* (REV)	5′-GAGCGACCAAAGGAACCATA-3′	Figures 1A,B, 3B,C,F, 4C,D and 5D
h-*18S* (FWD)	5′-ACCCGTTGAACCCCATTCGTG-3′	Figures 1C, 2A–C and 3D
h-*18S* (REV)	5′-GCCTCACTAAACCATCCAATC GG-3′	Figures 1C, 2A–C and 3D
h-*ACTB* (FWD)	5′-CACCAACTGGGAGGACAT-3′	Figure 1A
h-*ACTB* (REV)	5′-GCACAGCCTGGATAGCAAC-3′	Figure 1A
h-*ACTB* (FWD)	5′-CACCAACTGGGACGACAT -3′	Figure 2D
h-*ACTB* (REV)	5′-GCACAGCCTGGATAGCAAC -3′	Figure 2D
m-*Tnfrsf12a* (FWD)	5′-GTGTTGGGATTCGGCTTGGT-3′	Figures 1A,B, 3B,C, 4C,D and 5D

**Table 3:** Primers used in the study for ChIP qPCR.

Title	Sequence	Figure
m-*Tnfrsf12a* Promoter (FWD)	5′-TTTCCTTCGCTCCACATCGT-3′	Figure 4E
m-*Tnfrsf12a* Promoter (REV)	5′-ATTCAACACAGTCCCGCCAA-3′	Figure 4E

## Data Availability

Data are available from the corresponding authors and the first author upon request.
